# The Training of Sight

**Published:** 1900-11-10

**Authors:** 


### THE TRAINING OF SIGHT.

Lord Wolseley having lately remarked upon the good sight of the Boers as one cause at least of their good shooting, and having ascribed this good sight to its constant exercise in the open air, Mr. Brudenell Carter has pointed out that it is not merely a question of open air but of the training of the sight upon things that are far off and difficult to see. The defective vision possessed by so many children who have been brought up in towns is not caused by errors of refraction alone, common as these are, but by an actual deficiency in acuteness of vision, a lack of development in the nervous structures involved in the act of seeing. "Vision," he says, "like every other nerve function, must be cultivated for the attainment of a high degree of excellence. The visual power of London children is not cultivated by their environment. They see the other side of the street in which they live, and the carts and omnibuses of the thoroughfares. They scarcely ever have the visual attention directed strongly to any object which it is difficult to see, or which subtends a visual angle approaching the limits of visibility; and hence the seeing function is never exerted, or at least is not habitually exerted to anything like what should be the extent of its powers. With a country child the case is widely different." Mr. Brudenell Carter would like to see a place given to excellence of vision among the various physical qualifications which are habitually tested by competition and for which prizes are awarded, and he urges the desirability of volunteers taking up the exercise and training of the sight. "It is at least certain that our riflemen would not shoot worse for having learnt to see better."

